# Supplementary figures and images for: Empagliflozin reduces podocyte lipotoxicity in experimental Alport syndrome
Source: eLife. 2023 May 2;12:e83353. doi: 10.7554/eLife.83353 (PMC10185338; doi:10.7554/eLife.83353)

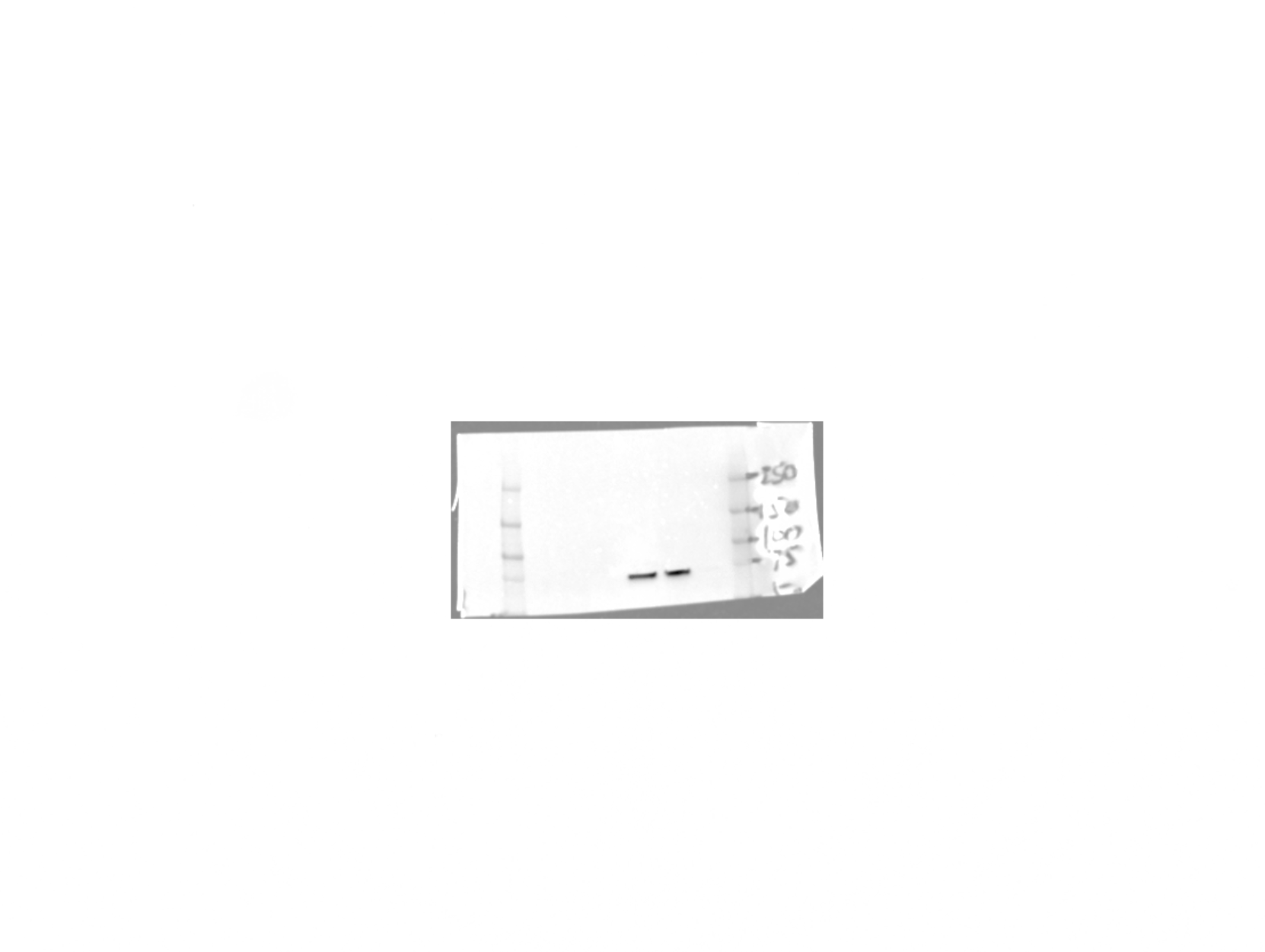

Supplement: Figure 1—source data 1. [file elife-83353-fig1-data1.zip › Figure 1-source data/Figure 1-source data 1.tif]

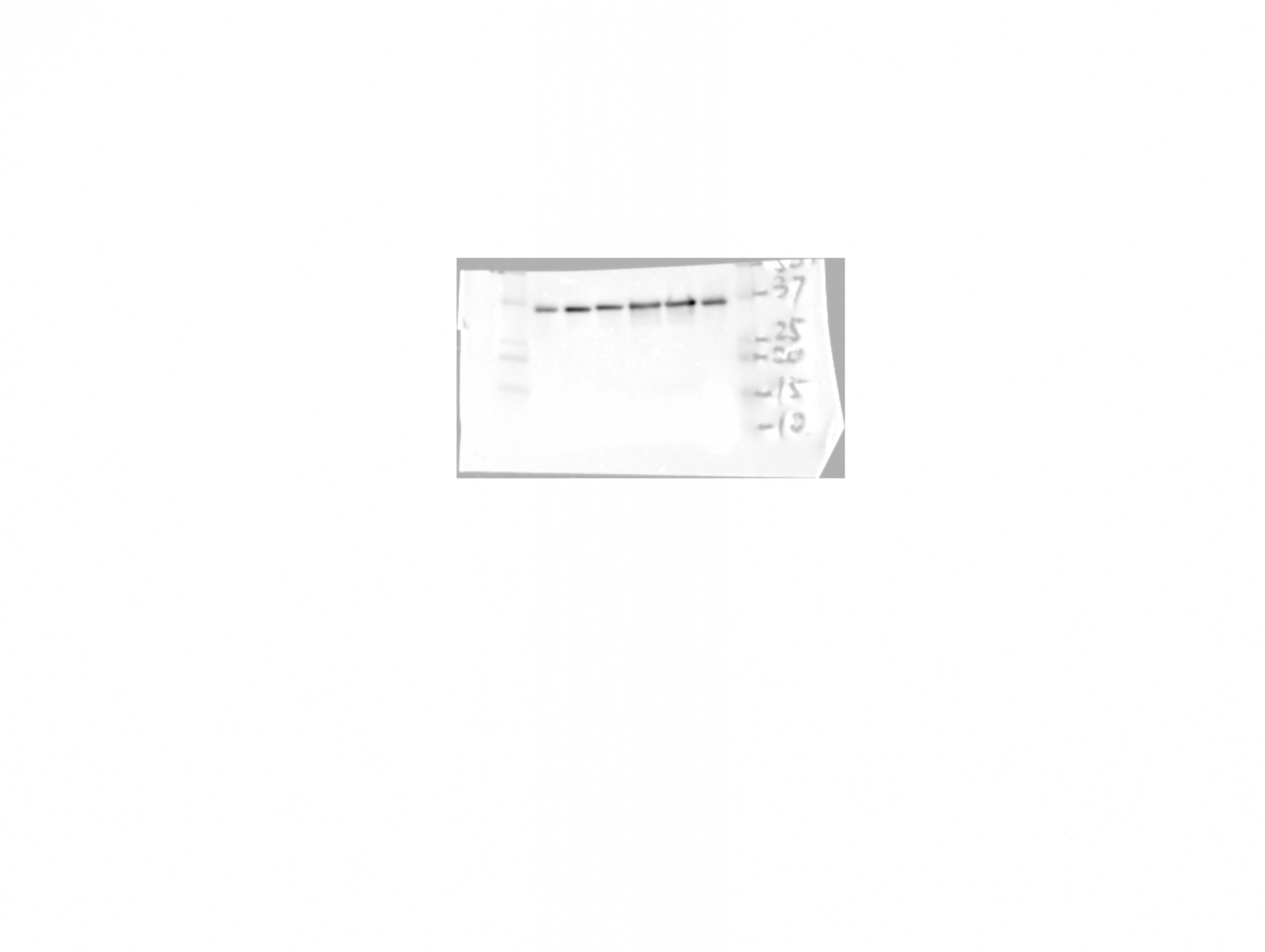

Supplement: Figure 1—source data 1. [file elife-83353-fig1-data1.zip › Figure 1-source data/Figure 1-source data 2.tif]

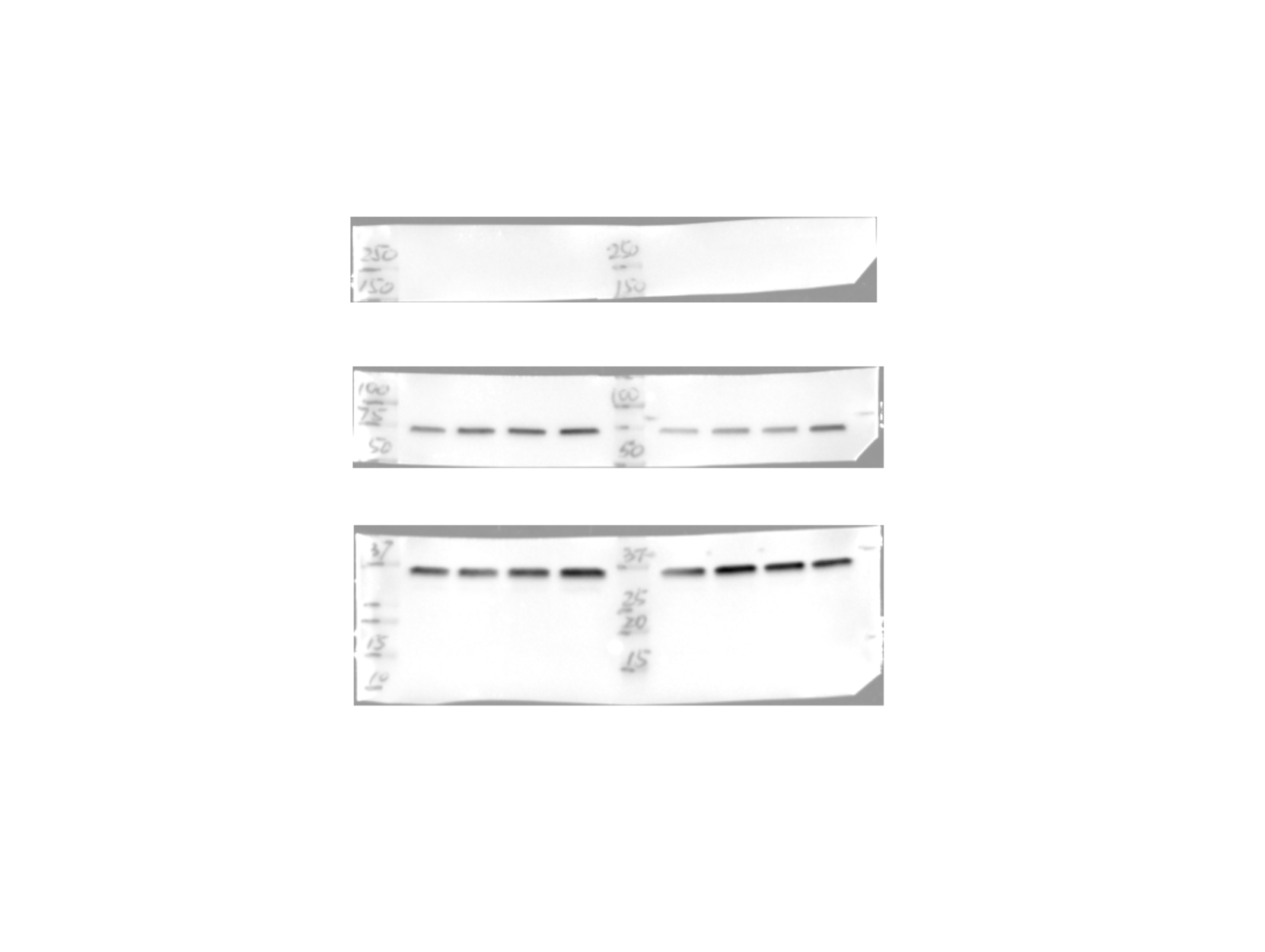

Supplement: Figure 1—source data 1. [file elife-83353-fig1-data1.zip › Figure 1-source data/Figure 1-source data 3.tif]

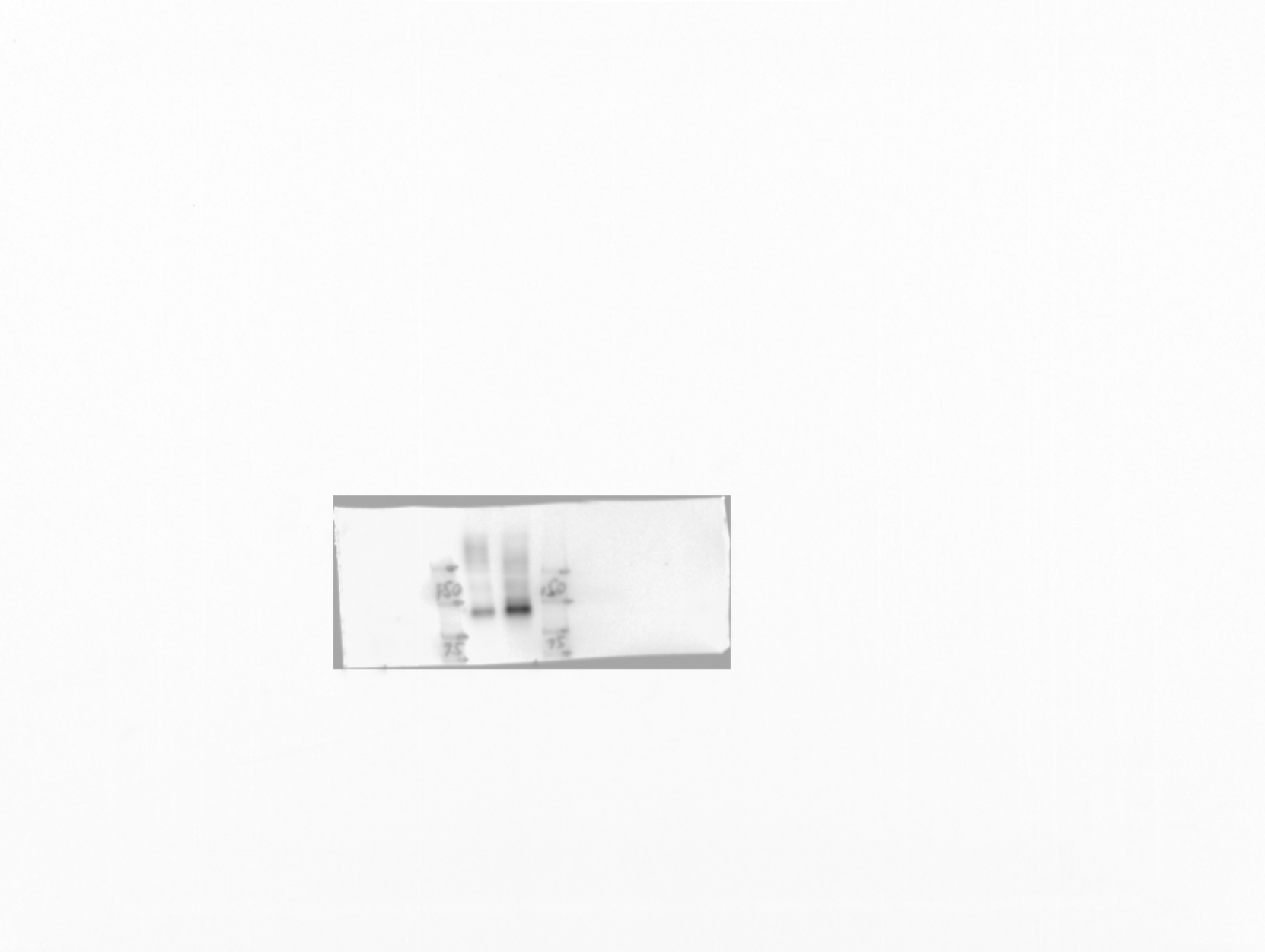

Supplement: Figure 1—figure supplement 1—source data 1. [file elife-83353-fig1-figsupp1-data1.zip › Figure 1-figure supplement 1-source data/Figure 1-figure supplement 1-source data 1.tif]

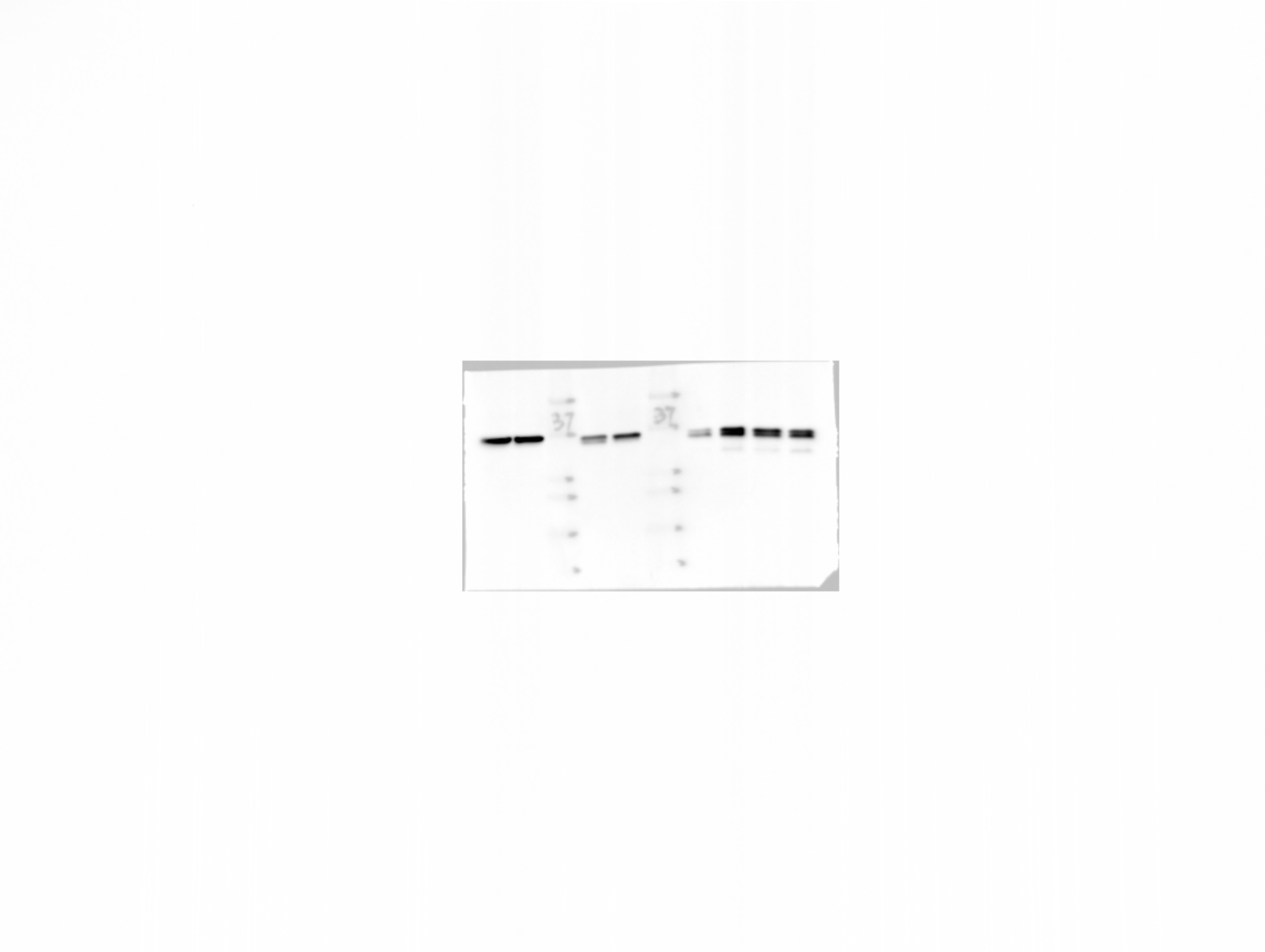

Supplement: Figure 1—figure supplement 1—source data 1. [file elife-83353-fig1-figsupp1-data1.zip › Figure 1-figure supplement 1-source data/Figure 1-figure supplement 1-source data 2.tif]

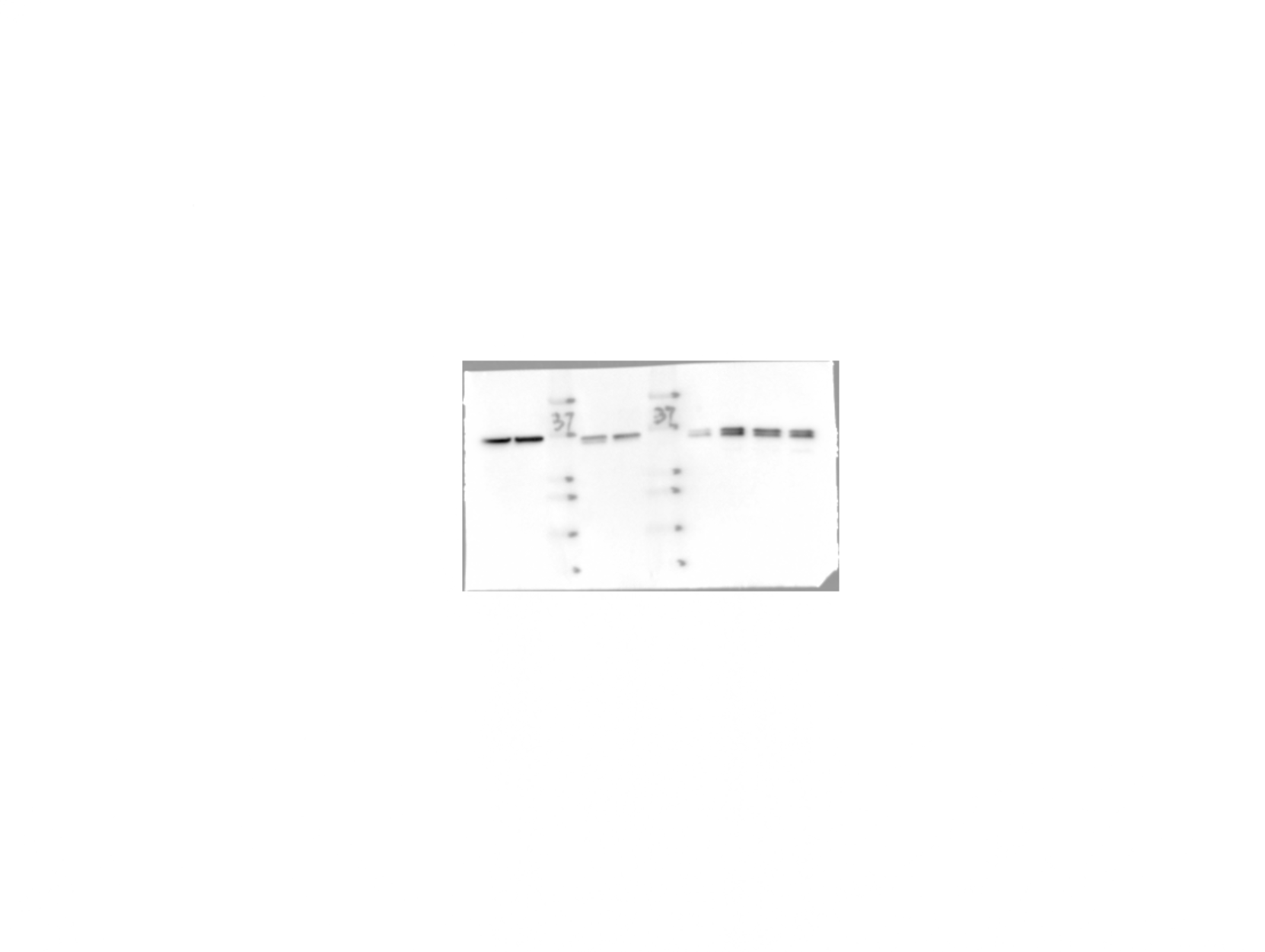

Supplement: Figure 1—figure supplement 1—source data 1. [file elife-83353-fig1-figsupp1-data1.zip › Figure 1-figure supplement 1-source data/Figure 1-figure supplement 1-source data 3.tif]

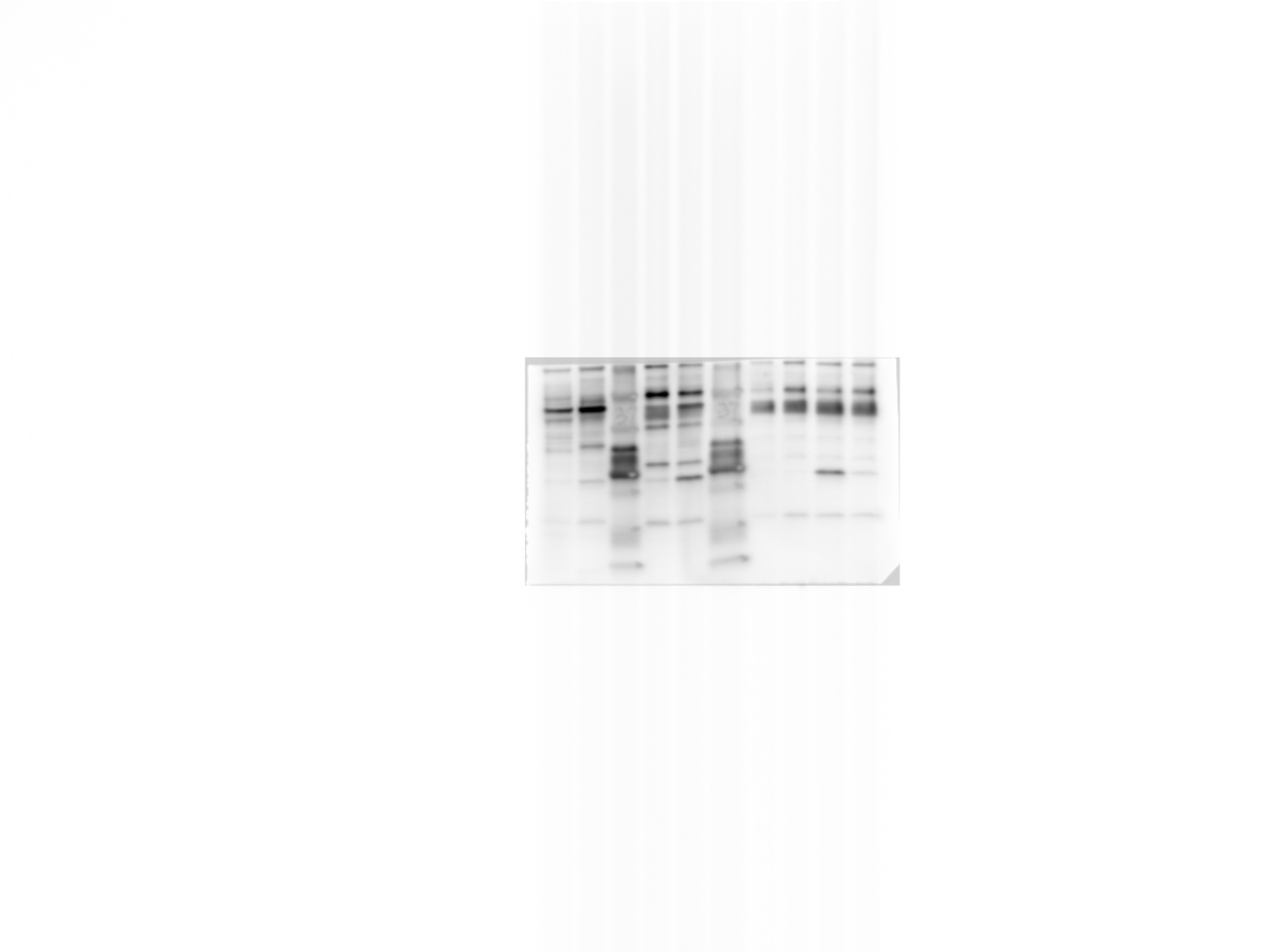

Supplement: Figure 1—figure supplement 1—source data 1. [file elife-83353-fig1-figsupp1-data1.zip › Figure 1-figure supplement 1-source data/Figure 1-figure supplement 1-source data 4.tif]

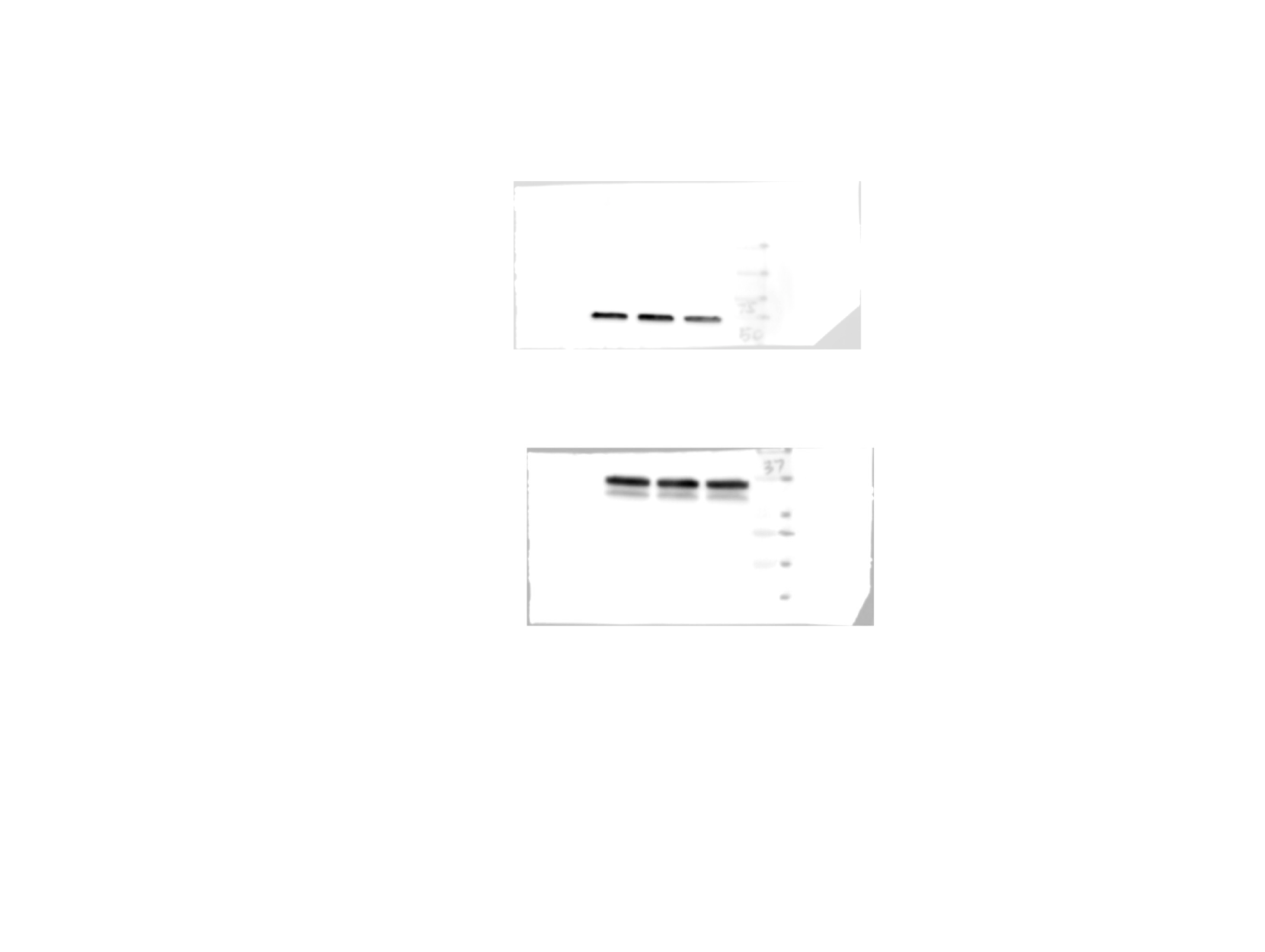

Supplement: Figure 4—source data 1. [file elife-83353-fig4-data1.zip › Figure 4-source data/Figure 4-source data 1.tif]

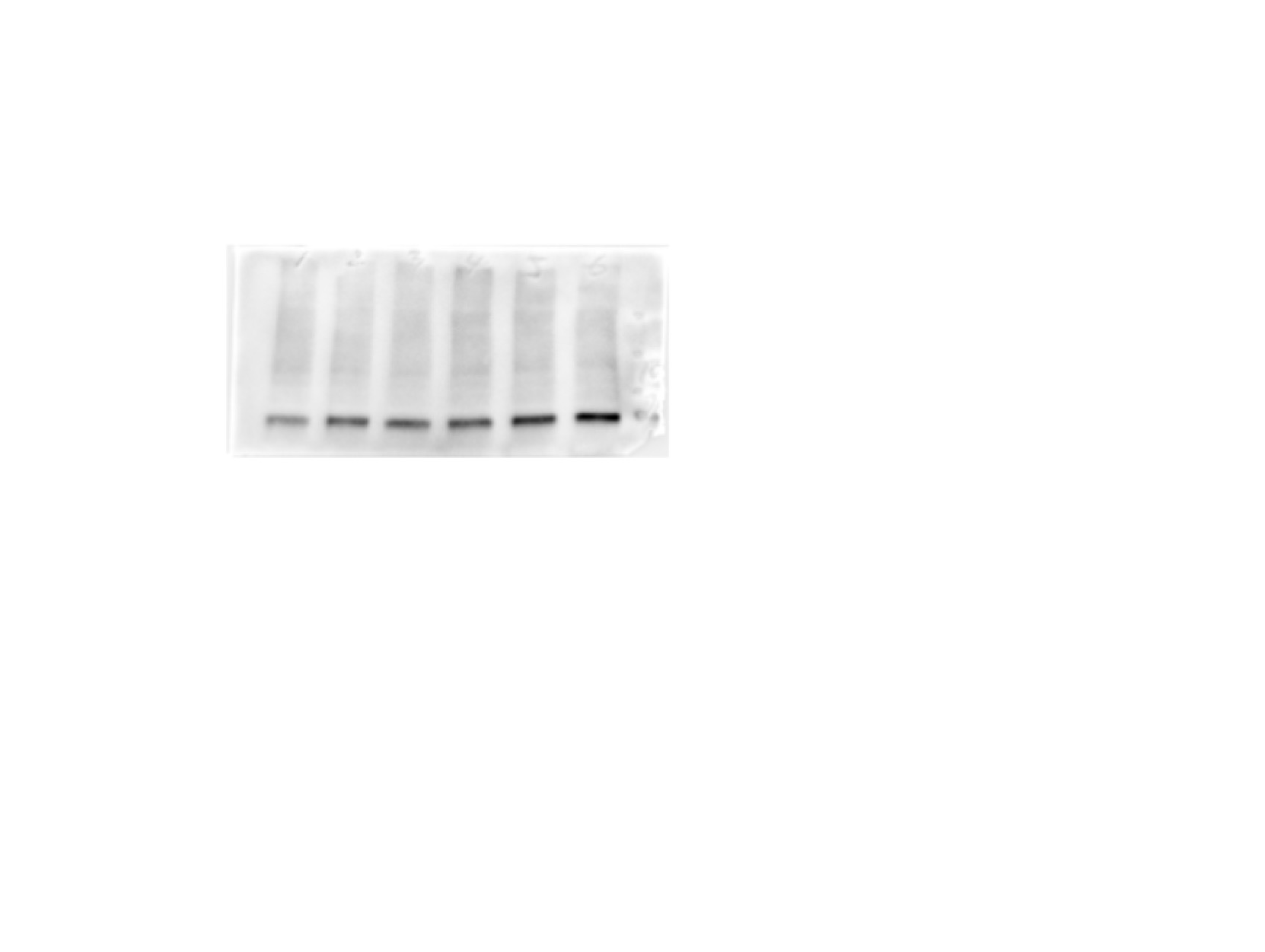

Supplement: Figure 4—source data 1. [file elife-83353-fig4-data1.zip › Figure 4-source data/Figure 4-source data 2.tif]

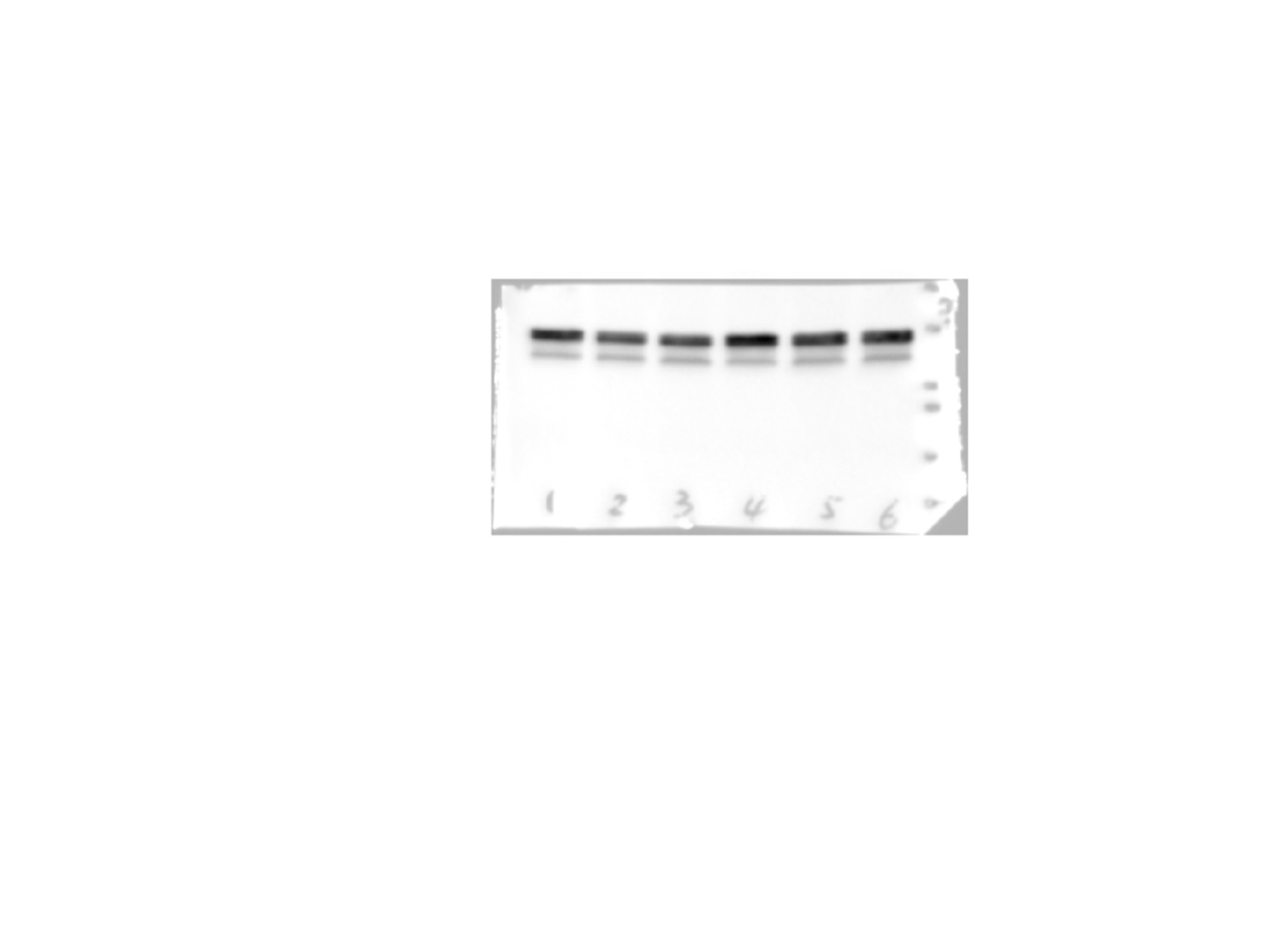

Supplement: Figure 4—source data 1. [file elife-83353-fig4-data1.zip › Figure 4-source data/Figure 4-source data 3.tif]

Figure 1B

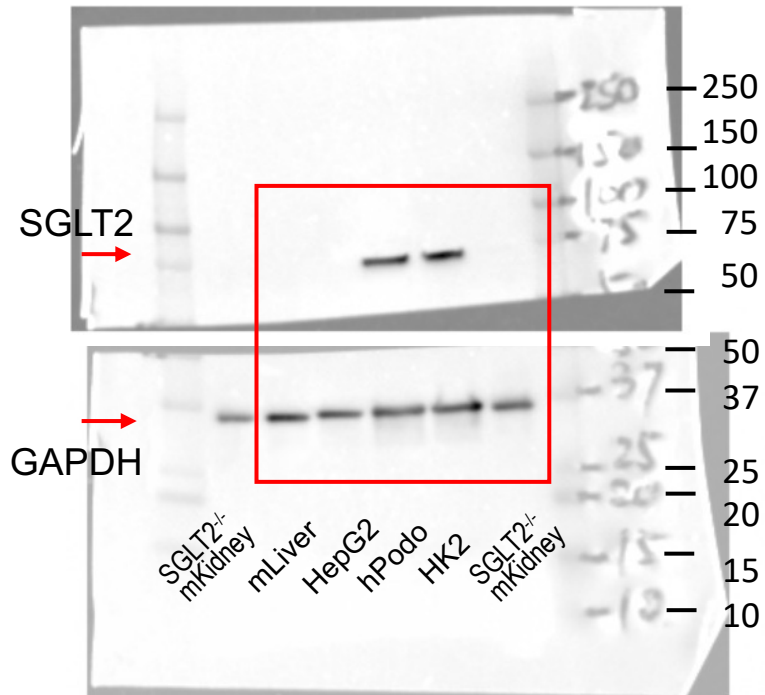

Figure 1C

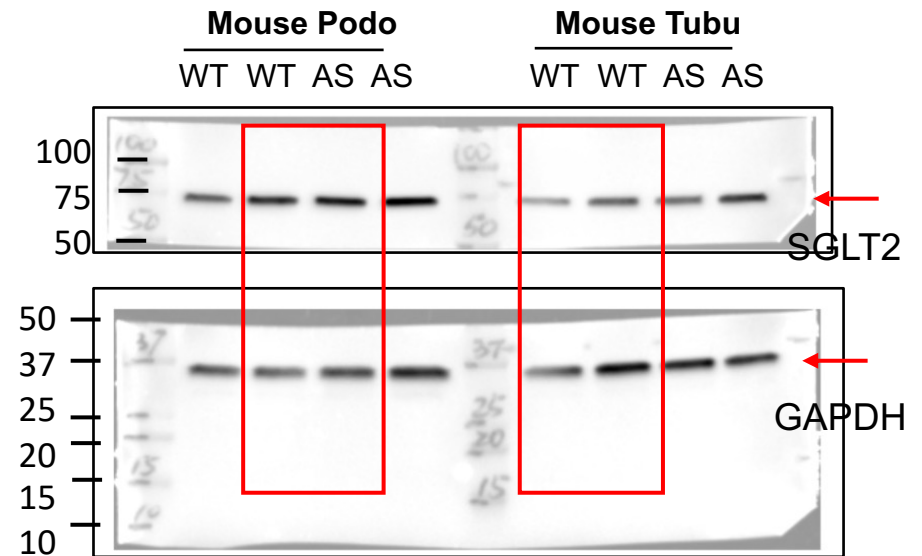

Figure 1-Figure supplement 1

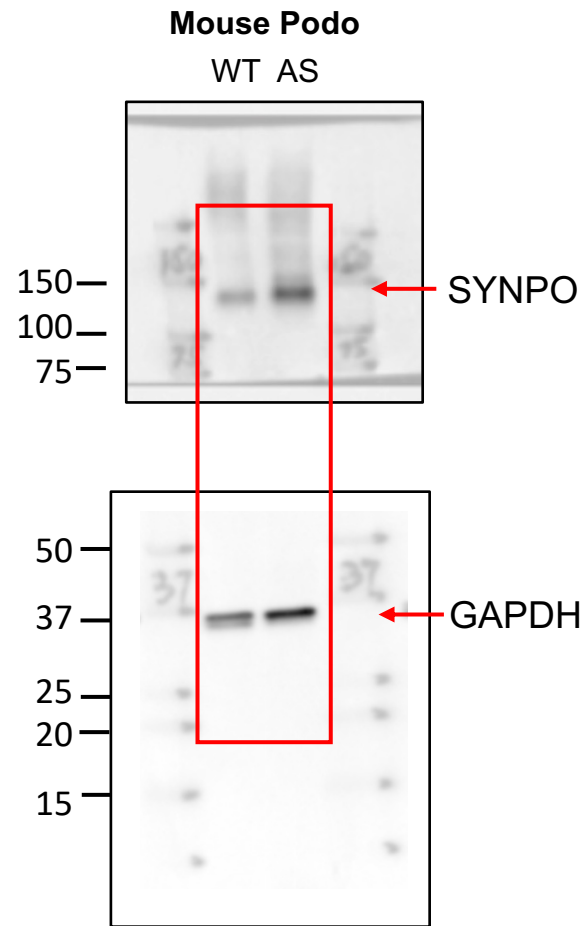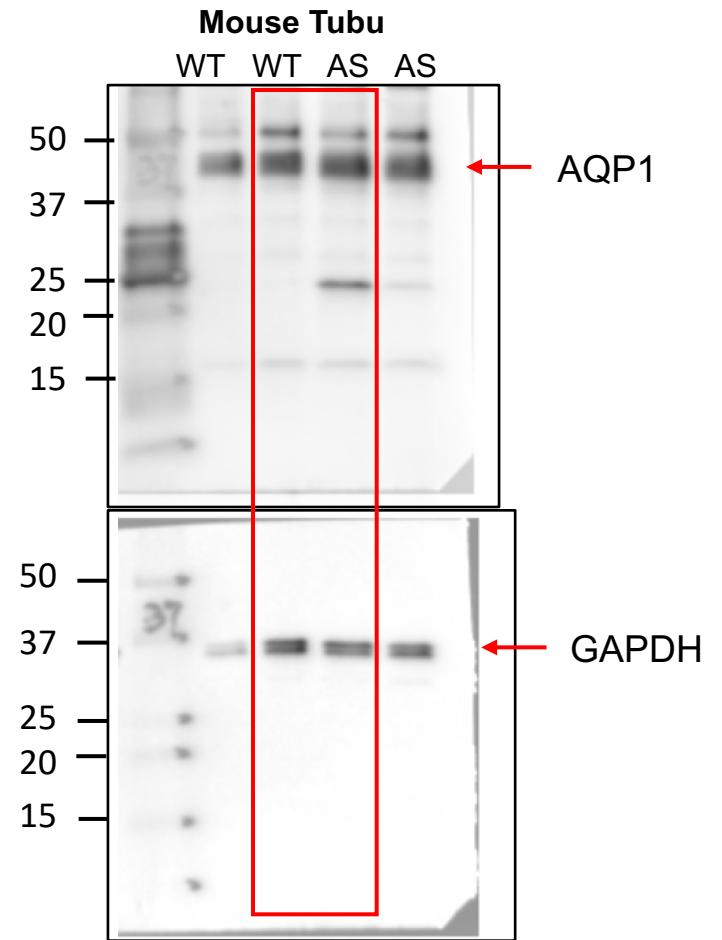

Figure 4A

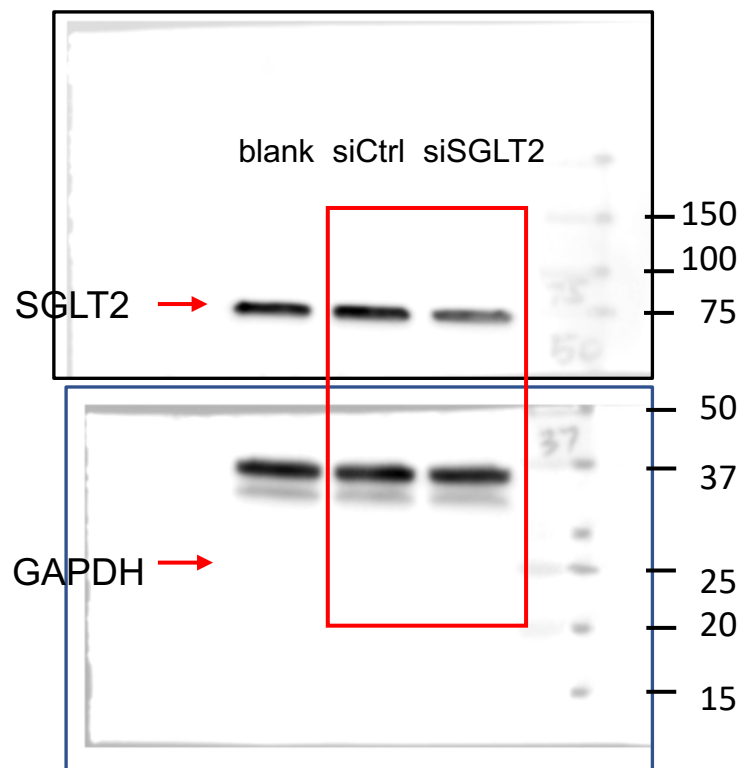

Figure 4E

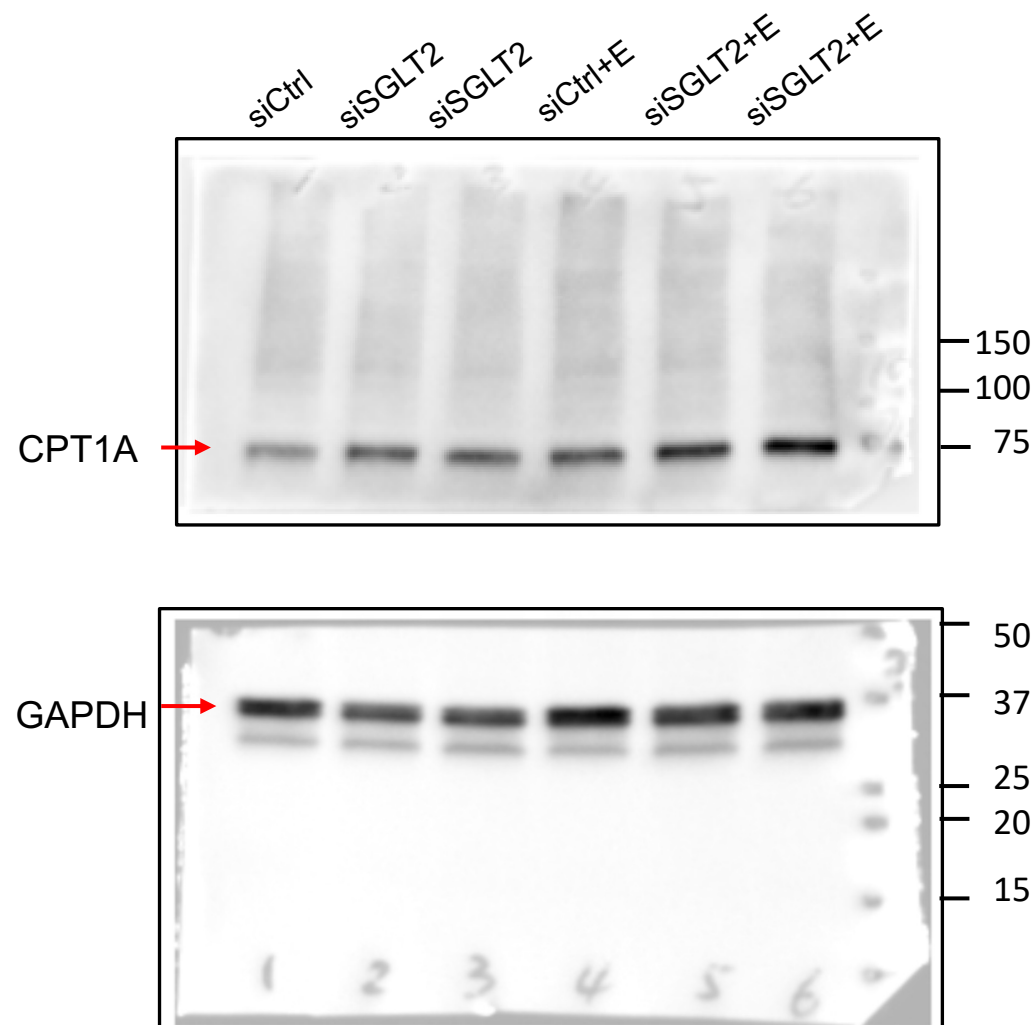

Supplement: Source data 1. [file elife-83353-data1.pdf]
